# The incidence and prevalence of cardiovascular diseases in gout: a systematic review and meta-analysis

**DOI:** 10.1007/s00296-021-04876-6

**Published:** 2021-05-13

**Authors:** Peter Cox, Sonal Gupta, Sizheng Steven Zhao, David M. Hughes

**Affiliations:** 1 Institute of Systems, Molecular and Integrative Biology, University of Liverpool, Biosciences Building, Crown Street, Liverpool, L69 7BE UK; 2 School of Medicine, University of Liverpool, Liverpool, UK; 3 Musculoskeletal Biology, Institute of Life Course and Medical Sciences, University of Liverpool, Liverpool, UK; 4 Department of Health Data Science, University of Liverpool, Liverpool, UK

**Keywords:** Gout, Cardiovascular disease, Prevalence, Meta-analysis

## Abstract

The aims of this systematic review and meta-analysis were to describe prevalence of cardiovascular disease in gout, compare these results with non-gout controls and consider whether there were differences according to geography. PubMed, Scopus and Web of Science were systematically searched for studies reporting prevalence of any cardiovascular disease in a gout population. Studies with non-representative sampling, where a cohort had been used in another study, small sample size (< 100) and where gout could not be distinguished from other rheumatic conditions were excluded, as were reviews, editorials and comments. Where possible meta-analysis was performed using random-effect models. Twenty-six studies comprising 949,773 gout patients were included in the review. Pooled prevalence estimates were calculated for five cardiovascular diseases: myocardial infarction (2.8%; 95% confidence interval (CI)s 1.6, 5.0), heart failure (8.7%; 95% CI 2.9, 23.8), venous thromboembolism (2.1%; 95% CI 1.2, 3.4), cerebrovascular accident (4.3%; 95% CI 1.8, 9.7) and hypertension (63.9%; 95% CI 24.5, 90.6). Sixteen studies reported comparisons with non-gout controls, illustrating an increased risk in the gout group across all cardiovascular diseases. There were no identifiable reliable patterns when analysing the results by country. Cardiovascular diseases are more prevalent in patients with gout and should prompt vigilance from clinicians to the need to assess and stratify cardiovascular risk. Future research is needed to investigate the link between gout, hyperuricaemia and increased cardiovascular risk and also to establish a more thorough picture of prevalence for less common cardiovascular diseases.

**Supplementary Information:**

The online version contains supplementary material available at 10.1007/s00296-021-04876-6.

## Introduction

Gout is an inflammatory crystal arthropathy characterised by hyperuricaemia and intra-articular monosodium urate crystal deposition. The prevalence of gout in adults has been increasing over time, with an estimated prevalence of 3.2% in the UK (5.2% in men and 1.3% in women) [1], 3.9% in the USA (5.2% in men and 2.7% in females) [2] and 3.8% in Taiwan (5.2% in men and 2.3% in women) [3]. It is characterised by acute attacks typically lasting up to 14 days and is associated with hyperuricaemia, purine-rich diets and increased alcohol consumption [4]. Urate is the final product of purine metabolism, a step catalysed by the xanthine oxidase enzyme making it a pharmacological target for agents such as allopurinol or febuxostat [5]. Elevated urate levels predispose to crystal precipitation in the synovial fluid. This in turn leads to recruitment of monocytes and macrophages to perform phagocytosis and release proinflammatory cytokines, resulting in a local inflammatory response causing swelling of the soft tissue and joint [5].

Both gout and subclinical hyperuricaemia are associated with adverse cardiovascular outcomes. Hyperuricaemia has been linked with an increased incidence of both coronary heart disease (CHD) and cerebrovascular accident (CVA) [6, 7]. Several studies have found gout to be associated with an increased risk of cardiovascular diseases, such as CHD and CVA, but the evidence is conflicting [8–11]. Furthermore, there have been no previous reviews assessing the prevalence of venous thromboembolism (VTE) in patients with gout.

The aims of this review were to: (1) describe the incidence and prevalence of cardiovascular disease in gout, (2) compare these results with non-gout controls and (3) consider whether there are differences according to geography.

## Methods

The review was performed in accordance with the Preferred Reporting Items for Systematic Reviews and Meta-Analyses (PRISMA) guidelines and registered as PROSPERO CRD42021232717 [12]. In January 2021, PubMed, Scopus and Web of Science were searched using the following MeSH terms and keywords: [Gout] AND [Cardiovascular OR Cardiovascular disease].

Studies were included if they reported an adult population with gout and recorded either the number of cases of a given cardiovascular disease or the incidence of cardiovascular disease per person years. Studies with non-representative sampling (e.g. all male participants), where a cohort had been used in another study, small sample size (< 100) and where gout could not be distinguished from other rheumatic conditions were excluded, as were reviews, editorials and comments. There was no restriction by study setting (e.g. primary care, secondary care, outpatients) or by country. Studies reporting interventional or secondary prevention trials were excluded.

Titles and abstracts were screened for eligibility. Sample size and the prevalence of the investigated cardiovascular diseases were extracted from each study, alongside demographic data, the data source, how they defined gout and the outcome and comparisons with non-gout controls. Quality assessment was performed using a modified version of the Newcastle–Ottawa Scale. Studies were scored based on 4 aspects: representativeness (0–2), sample size (0–1), gout definition (0–1) and ascertainment of cardiovascular disease (0–1). A higher score indicated better methodological quality and lower risk of bias. The review was conducted by one author (PC) and a second performed a 10% validation (SG).

Where prevalence data was reported on a cardiovascular condition in ≥ 3 cohorts, meta-analysis was performed. Pooled prevalence was calculated using random-effect models (DerSimonian-Laird) and heterogeneity presented using the *I*^2^ statistic. Funnel plots were produced to assess risk of publication bias. The meta-analysis was performed using R version 4.0.3.

## Results

**Fig. 1:**
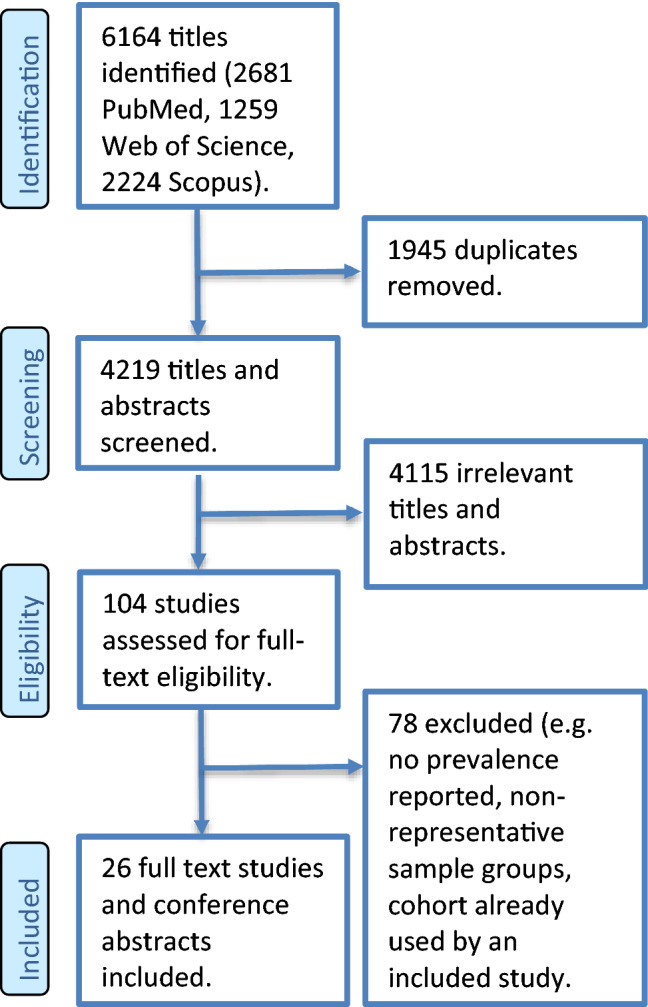
Study selection flowchart

The search produced 6164 publications, of which 26 were included in the review after duplicates and exclusions, illustrated in Fig. 1. There was a total of 949,773 patients with gout, although one study by Singh et al. did not report the sample size of gout patients alone [13]. Eleven studies investigated populations from the USA, followed by 6 from Taiwan, 4 from the Netherlands, 3 from the UK, 1 from China and 1 from Canada.

The mean age ranged from 52.5 to 80.1, with a medium value of 62.4, and the percentage of male participants from 60.4% to 99.1%. Twelve of the studies involved participants at primary care or population level while the remaining 14 examined individuals from hospital or outpatients’ settings. The study by Seminog et al. analysed 2 different cohorts comprising hospitalised patient’s records: the record-linked Hospital Episode Statistics (HES) dataset for England from 1999 to 2011 and the Oxford Record Linkage Study (ORLS) dataset from 1963 to 1998 [10].

The majority of papers, *n* = 16, defined gout by diagnostic code, predominantly International Classification of Diseases, with 4 accepting self-reported gout, 2 according to the Wallace Criteria, 1 by the International Classification of Primary Care (ICPC), 1 physician diagnosed, 1 by attending rheumatic outpatients and 1 by crystal-proven joint fluid analysis. Most bias scores were 3 out of a potential 6 stars (Supplementary Table S1 and Fig S1), indicating moderate bias.

Myocardial infarction (MI) was studied in eight papers, the most frequently investigated cardiovascular disease, followed by VTE investigated in six studies. Cardiovascular disease was also predominantly defined by diagnostic code *n* = 18, with 3 studies requiring a physician diagnosis, 2 by transthoracic echocardiogram and 1 each by a cardiologist reviewing resting ECGs, by the ICPC and by self-reporting The complete data extraction is provided in Supplementary Table S2.

### Prevalence of cardiovascular diseases

**Fig. 2:**
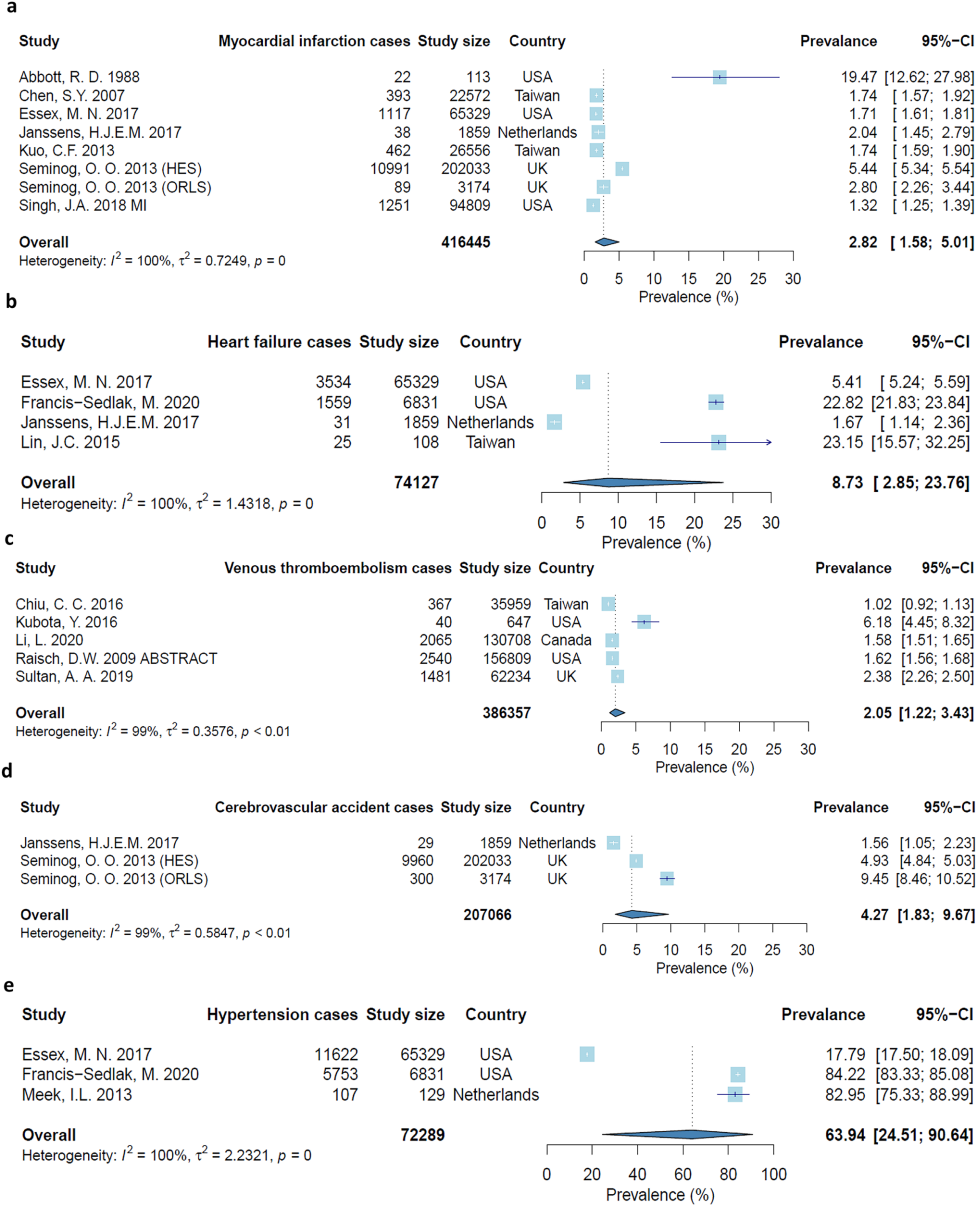
Forest plots of pooled prevalence of: **a** 2.8% for myocardial infarction, **b** 8.7% for heart failure, **c** 2.1% for venous thromboembolism, **d** 4.3% for cerebrovascular accident and **e** 63.9% for hypertension

Pooled prevalence estimates were calculated for five cardiovascular diseases, of which hypertension had the highest prevalence of 63.9% (24.5%, 90.6%) followed by heart failure with 8.7% (2.9%, 23.8%), CVA with 4.3% (1.8, 9.7), MI with 2.8% (1.6, 5.0) and VTE with 2.1% (1.2, 3.4). The forest plots for each are shown in Fig. 2 and funnel plots provided in Supplementary Fig S2–S6. There was significant heterogeneity in the meta-analysis, with *I*^2^ ≥ 99% throughout.

The full results for prevalence and incidence are listed in Table 1. MI prevalence ranged from 1.3% to 19.5%, with the largest study of 202,033 gout patients recording prevalence at 5.4%. This was the largest study included in the review and also reported CVA prevalence at 4.9%. Incidence of CVA was measured at 9.3 cases per 1000 person years and also as 7.5 in males and 13.7 in females. Heart failure prevalence ranged from 1.7% to 23.2%. A small study of 647 gout patients found VTE prevalence to be 6.2% while the remaining studies were much larger and measured values between 1.0% and 2.4%. The largest study investigating hypertension comprised more than 65,000 gout patients and reported prevalence of 17.8%, compared with two other smaller studies which measured prevalence at above 80%.

**Table 1:** Summary of studies included in the systematic review

Study	Country	Source	Sample Size	Prevalence	Incidence per person 1000 years
Myocardial infarction					
Abbott et al. [8]	USA	Framingham Study	113	19.47	
Chen et al. [14]	Taiwan	Ho-Ping Gout Database, inpatients	22,572	1.74	
Essex et al. [15]	USA	Humedica database	65,329	1.71	
Janssens et al. [16]	Netherlands	GP Database	1859	2.04	
Kuo et al. [3]	Taiwan	Taiwanese National Health Insurance database	26,556	1.74	2.2
Seminog et al. (HES) [10]	UK	Hospital Episode Statistics for England	202,033	5.44	
Seminog et al. (ORLS) [10]	UK	Oxford Record Linkage Study	3174	2.8	
Singh et al. MI [17]	USA	Medicare 5% claims data	94,809	1.32	4.1
Clarson et al. [18]	UK	Clinical Practice Research Datalink	8386		M 9.27, F 6.11
Cerebrovascular accident					
Janssens et al. [16]	Netherlands	GP Database	1859	1.56	
Seminog et al. (HES) [10]	UK	Hospital Episode Statistics for England	202,033	4.93	
Seminog et al. (ORLS) [10]	UK	Oxford Record Linkage Study	3174	9.45	
Clarson et al. [18]	UK	Clinical Practice Research Datalink	8386		M 7.45, F 13.71
Colantonio et al. [11]	USA	REGARDS cohort	187		9.3
Heart failure					
Essex et al. [15]	USA	Humedica database	65,329	5.41	
Francis-Sedlak et al. [19]	USA	Humanan Research Database	6831	22.82	
Janssens et al. [16]	Netherlands	GP Database	1859	1.67	
Lin et al. [20]	Taiwan	N/A	108	23.15	
Colantonio et al. [11]	USA	REGARDS cohort	187		13.1
Venous thromboembolism					
Chiu et al. [21]	Taiwan	Taiwanese National Health Insurance database	35,959	1.02	1.348
Kubota et al. [22]	USA	Atherosclerosis Risk in Communities Study	647	6.18	
Raisch et al. ABSTRACT [23]	USA	Veterans Affairs national database	156,809	1.62	
Li et al. [24]	Canada	Population Data BC Database	130,708	1.58	2.63
Sultan et al. [25]	UK	Primary Care and NHS Hospital databases	62,234	2.38	3.73
Huang et al. [26]	Taiwan	National Health Insurance Research database	57,981		0.526
Cardiovascular mortality					
Disveld et al. [27]	Netherlands	Gout Arnhem-Liemers Cohort	700		7.71
Kuo et al. [28]	Taiwan	Health screening programme in Chang Gung Memorial Hospital	1311		2.09
Stack et al. [29]	USA	Third National Health and Nutrition Examination Survey	468		23.1
Hypertension					
Essex et al. [15]	USA	Humedica database	65,329	17.79	
Francis-Sedlak et al. [19]	USA	Humanan Research Database	6831	84.22	
Meek et al. [30]	Netherlands	Arthritis Center Twente (outpatients) and the Doetinchem Cohort	129	83	
Pan et al. [31]	China	Singapore Chinese Health Study	573		52.54
Angina					
Abbott et al. [8]	USA	Framingham Study	113	20.35	
Janssens et al. [16]	Netherlands	GP Database	1859	0.97	
Clarson et al. [18]	UK	Clinical Practice Research Datalink	8386		M 11.80, F 12.32
Transient ischaemic attack					
Janssens et al. [16]	Netherlands	GP Database	1859	0.81	
Clarson et al. [18]	UK	Clinical Practice Research Datalink	8386		M 6.04, F 9.34
Atrial fibrillation					
Francis-Sedlak et al. [19]	USA	Humanan Research Database	6831	21.48	
Kim et al. [32]	USA	United Health Care database	70,015	1.47	7.19
Singh et al. AF [13]	USA	Medicare 5% claims data	N/A		43.4
Peripheral vascular disease					
Janssens et al. [16]	Netherlands	GP Database	1859	1.51	
Clarson et al. [18]	UK	Clinical Practice Research Datalink	8386		M 5.60, F 7.09
Any coronary heart disease					
Clarson et al. [18]	UK	Clinical Practice Research Datalink	8386		M 28.46, F 9.11
Colantonio et al. [11]	USA	REGARDS cohort	187		16.0
Aortic stenosis					
Chang et al. [33]	USA	Outpatients	112	21.43	
Aortic aneurysm					
Janssens et al. [16]	Netherlands	GP Database	1859	0.91	
Cardiovascular disease					
Janssens et al. [34]	Netherlands	Continuous Morbidity Registration	170	25.88	

*F* Female, *HES* Hospital Episode Statistics, *M* Male, *ORLS* Oxford Record Linkage Study

Of the remaining diseases there was a great range in reported prevalence, from 25.9% for general cardiovascular disease to 0.8% for transient ischaemic attack. Likewise, the highest incidence rate reported was that of 43.4 for atrial fibrillation, the lowest being 2.1 reported in cardiovascular mortality. There was some variation amongst rates for individual diseases, such as cardiovascular mortality incidence reported at both 23.1 and 2.1.

### Prevalence compared with controls

Of the 26 studies, 16 reported comparisons with non-gout controls. These have mainly reported as adjusted hazard ratio (HR), but also as adjusted odds ratio (OR), adjusted relative risk (RR) and standardised mortality rate, with different studies measuring different outcomes such as incidence and prevalence. These results are reported in Table 2, with the general trend being that of increased risk in the gout group, particularly for MI. A small number of results indicate a risk decrease, but for each the confidence intervals cross 1.00, so can be deemed statistically insignificant.

**Table 2:** Studies comparing results between gout patients and non-gout controls

Cardiovascular disease	Study	Measure of effect	Effect size	95% confidence interval
Myocardial infarction	Clarson et al. [18]	HR for MI risk adjusted for age, sex, BMI, smoking status, alcohol, Charlson comorbidity index, comorbidities and medication	**M 1.12**, F 0.97	M 1.00–1.27, F 0.77–1.22
	Seminog et al. (HES) [10]	RR for MI adjusted for age, sex, time, area of residence and deprivation	**1.82**	1.78–1.85
	Seminog et al. (ORLS) [10]	RR for MI adjusted for age, sex, time, area of residence and deprivation	**1.95**	1.57–2.40
	Kuo et al. [3]	HR for incidence adjusted for age, sex and comorbidities	**1.23**	1.11–1.36
	Singh et al. MI [17]	HR for incidence adjusted for age, sex, comorbidities and medication	**2.08**	1.95–2.21
Cerebrovascular accident	Clarson et al. [18]	HR for CVA risk adjusted for age, sex, BMI, smoking status, alcohol, Charlson comorbidity index, comorbidities and medication	M 0.93, **F 1.34**	M 0.81–1.06, F 1.15–1.57
	Seminog et al. (HES) [10]	RR for CVA adjusted for age, sex, time, area of residence and deprivation	**1.71**	1.68–1.75
	Seminog et al. (ORLS) [10]	RR for CVA adjusted for age, sex, time, area of residence and deprivation	**1.91**	1.70–2.14
	Colantonio et al. [11]	HR for prevalence adjusted for age, sex, race, region of residence, income, education, alcohol, smoking, BMI, physical activity, dietary patterns, comorbidities and medication	0.83	0.48–1.43
Venous thromboembolism	Chiu et al. [21]	HR for DVT risk	**1.38**	1.18–1.62
	Sultan et al. [25]	HR for VTE risk adjusted for age, sex, BMI, alcohol, smoking, time, deprivation, hospital admission and medication	**1.25**	1.15–1.35
	Kubota et al. [22]	HR for VTE risk adjusted for age, sex, race, BMI, smoking and comorbidity	1.33	0.95–1.86
	Huang et al. [26]	HR for incidence adjusted for age, sex and comorbidities	**1.66**	1.37–2.01
	Li et al. [24]	HR for incidence adjusted for age, sex, healthcare utilisation, Charlson comorbidities index, comorbidities and medications	**1.22**	1.13–1.32
Heart failure	Colantonio et al. [11]	HR for prevalence adjusted for age, sex, race, region of residence, income, education, alcohol, smoking, BMI, physical activity, dietary patterns, comorbidities and medication	**1.97**	1.22–3.19
Cardiovascular mortality	Stack et al. [29]	HR for prevalence adjusted for age, sex, race, BMI and comorbidities	**1.58**	1.13–2.19
	Disveld et al. [27]	Standardized mortality rate	**6.75**	4.64–8.86
Atrial fibrillation	Kim et al. [32]	HR for incidence adjusted age, sex, comorbidities, medication and healthcare utilisation	**1.21**	1.11–1.33
	Singh et al. AF [13]	HR for incidence adjusted for age, sex and medication	**1.92**	1.88–1.96
Angina	Clarson et al. [18]	HR for angina risk adjusted for age, sex, BMI, smoking status, alcohol, charlson comorbidity index, comorbidities and medication	M 1.02, **F 1.28**	M 0.92–1.13, F 1.09–1.51
Transient ischaemic attack	Clarson et al. [18]	HR for transient ischaemic attack risk adjusted for age, sex, BMI, smoking status, alcohol, charlson comorbidity index, comorbidities and medication	M 1.02, **F 1.26**	M 0.88–1.18, F 1.05–1.53
Peripheral vascular disease	Clarson et al. [18]	HR for peripheral vascular disease risk adjusted for age, sex, BMI, smoking status, alcohol, charlson comorbidity index, comorbidities and medication	**M 1.18, F 1.89**	M 1.01–1.38, F 1.50–2.38
Hypertension	Pan et al. [31]	HR for hypertension risk adjusted for age, sex, year, education, BMI, alcohol, smoking, physical activity and diabetes	**1.18**	1.02–1.37
	Meek et al. 2013 [30]	OR for prevalence adjusted for age and sex	**2.7**	1.7–4.3
Any coronary heart disease	Clarson et al. [18]	HR for coronary heart disease risk adjusted for age, sex, BMI, smoking status, alcohol, charlson comorbidity index, comorbidities and medication	**M 1.08, F 1.25**	M 1.01–1.15, F 1.12–1.39
	Colantonio et al. [11]	HR for prevalence adjusted for age, sex, race, region of residence, income, education, alcohol, smoking, BMI, physical activity, dietary patterns, comorbidities and medication	1.21	0.79–1.84
Aortic stenosis	Chang et al. [33]	OR for prevalence matched for age	**2.08**	1.00–4.32

Bold text indicates statistical significance

*BMI* Body mass index, *DVT* Deep vein thrombosis, *HES* Hospital Episode Statistics, *HR* Hazard ratio, *OR* Odds ratio, *ORLS* Oxford Record Linkage Study, *RR* Relative risk

### Prevalence of cardiovascular diseases by geography

Seven cardiovascular diseases were studied in three or more countries. The highest prevalence of MI was reported in the USA at 19.5% (12.6, 28.0), followed by the UK at 5.4% (5.3, 5.5). Other studies investigating the Netherlands, Taiwan and the USA had similar rates, including 2.0% (1.5, 2.8), 1.7% (1.6, 1.9) and 1.7% (1.6, 1.8) respectively. Additionally, the incidence rate per 1000 person years in the UK was higher than that of the USA and Taiwan, at 9.3 and 6.1 for males and females respectively in the UK compared with 4.1 for the USA and 2.2 for Taiwan.

The prevalence of CVA followed a similar trend with 9.5% (8.5, 10.5) and 4.9% (4.8, 5.0) reported in the UK compared with 1.6% (1.1, 2.2) in the Netherlands. For VTE, the USA recorded the highest prevalence of 6.2% (4.5, 8.3). Prevalence in the UK and Taiwan was measured at 2.4% (2.3, 2.5) and 1.0% (0.9, 1.1) respectively. The highest incidence rate per 1000 person years was seen in the UK at 3.7, followed by Canada at 2.6 and then Taiwan measured at both 1.4 and 0.5. The prevalence of heart failure was greatly varied measured at 23.2% (15.6, 32.3) in Taiwan, both 22.8% (21.8, 23.8) and 5.4% (5.2, 5.6) in the USA and 1.7% (1.1, 2.4) in the Netherlands.

Conditions studied in 2 or less countries were not analysed according to geography.

## Discussion

This systematic review and meta-analysis combined results from 26 studies and almost one million patients with gout to show that the prevalence of MI is 2.8%, heart failure is 8.7%, VTE is 2.1%, CVA is 4.3% and hypertension is 63.9%. The risk of cardiovascular diseases is higher in gout patients when compared with non-gout controls, as is the rate of cardiovascular mortality.

### Myocardial infarction

The pooled prevalence estimate for MI of 2.8% is comparable to 3.1% in rheumatoid arthritis [35], 3.2% in psoriatic arthritis [36] and 2.2% in axial spondyloarthritis [37]. There is an increased risk of MI compared to non-gout controls. There may be numerous explanations for this finding. Classical cardiovascular risk factors such as obesity, diabetes mellitus and hypertension have been shown to be more prevalent among those with gout [35]. There remains an association between gout and cardiovascular disease after adjusting for these factors, illustrating that gout conveys its own independent risk, potentially as a result of intermittent and chronic inflammation [38]. Even in patients without traditional risk factors, the risk of MI was found to be high, leading to the suggestion that gout is an early manifestation of metabolic abnormalities [3]. It has also been suggested that peripheral joint inflammation produces a greater systemic inflammatory response which may contribute to the increased prevalence of cardiovascular conditions [36]. While the underlying pathological mechanism remains unclear, this data indicates the effect of gout on cardiovascular disease has been underestimated for some time and needs reconsideration.

### Heart failure

The pooled prevalence value for heart failure of 8.7% was produced from results from individual studies which ranged from 1.7% to 23.2%, reflected in the broad 95% confidence interval of 2.9–23.8. These varying results may be attributable to differences in methodology, for example the prospective study by Lin et al. which investigated patients who both had gout and had undergone a transthoracic echocardiogram [20]. As this is not a routine investigation for patients with gout, it may well have increased the likelihood of this population having left ventricular dysfunction as the study reported. Furthermore, gout is predominantly managed in primary care. This means this cohort potentially suffered from more severe gout, or were more complex patients who required secondary care, both of which may increase the chances of comorbidities being present. The other study to report a large prevalence was by Francis-Sedlak et al., potentially stemming from a strict eligibility criteria which included just 6831 of 539,802 identified gout patients [19]. Enrolment in the database 6 months before and after diagnosis, 90 days of continuous urate lowering therapy and 2 subsequent serum uric acid measurements were required for inclusion. Comparable figures for other rheumatic conditions include 1.6% for rheumatoid arthritis [39], 1.3% for psoriatic arthritis [36], and 1.8 for axial spondyloarthritis [37] which may be closer to the true value.

The same argument of limited study groups and broad confidence intervals could also be made for the pooled prevalence for CVA of 4.3% (1.8%, 9.7%) and hypertension 63.9% (24.5, 90.6). The inability to determine a more precise estimate of prevalence may reflect a greater failing by the medical community to investigate this association, particularly given the potentially fatal nature of stroke.

### Venous thromboembolism

This is the first meta-analysis of VTE prevalence in gout populations which found a pooled prevalence of 2.1%, with most studies reporting increased risk compared with non-gout controls. VTE has also been shown to have an increased risk in other types of inflammatory arthritis [40, 41]. This predisposition to coagulation may be the result of inflammatory damage to the vascular endothelium [24]. It has been proposed that activation of the nucleotide-binding domain, leucine-rich-containing family, pyrin domain-containing-3 (NLRP3) inflammasome, which in turn stimulates release of interleukin-1β, could enhance this inflammatory response [42]. An idea to resolve this could be the long-term management of gout involving a serum urate target. This is a controversial topic, with questions over the number needed to treat and what the impacts, if any, of a long-term lowered serum urate would be [42].

### Cardiovascular mortality

All studies found the risk of cardiovascular mortality to be increased when compared with non-gout controls. A study by Kok et al. highlighted a finding that gout conferred a protective effect on cardiovascular mortality in those with chronic kidney disease [43]. In explaining this finding, the question is raised that perhaps it is not gout but rather urate-lowering therapies such as allopurinol that is the source of the reduction in cardiovascular risk. In hyperuricaemic patients, allopurinol has been associated with a reduced rate of all-cause mortality (HR 0.78; 95% CI 0.67, 0.91) [44], and of major cardiovascular events (HR 0.89; 95% CI 0.81, 0.97) [45]. A small-scale prospective randomized trial of patients with chronic kidney disease found that allopurinol compared to treatment as usual reduced both the risk of cardiovascular events and of hospitalisation [46]. However, in contrast to this a cohort study from Taiwan in a gout population did not observe any beneficial effect from allopurinol on cardiovascular risk [47], indicating the need for further research into the cardiovascular effect of allopurinol on patients with gout.

### Hypertension

Hypertension pooled prevalence was calculated at 63.9%, a finding which merits careful thought as hypertension is an important risk factor for the majority of the cardiovascular diseases mentioned in this review. This finding is derived from just 3 studies and may demonstrate a lack of quality evidence in this broader aspect of gout management. Evaluating these studies individually, the study by Francis-Sedlak et al. employed a strict inclusion criteria as previously mentioned, while the study by Meek et al. investigated a small sample size from a rheumatology outpatients department which implemented routine cardiovascular screening, potentially increasing the chance of detecting hypertension [19, 30]. The results of these studies vary considerably compared to that of Essex et al., which examined a much larger gout cohort at a population level [15]. Previously, uric acid has been shown to stimulate vascular smooth muscle cell proliferation in vitro, as well as both angiotensinogen and angiotensin II production [48]. A link has also been detected between hyperuricaemia and hypertension in animal models, noting elevated renin expression suggesting the underlying mechanism involves the renin-angiotensin system [49]. These studies provide plausibility for our finding of a very high hypertension prevalence in gout patients. Beyond MI, CVA and VTE there have been few studies looking at each condition and on occasion they report conflicting findings. This has thus far made it difficult to draw a conclusive impression on the effect of gout on cardiovascular disease prevalence for some of the less common conditions.

### Prevalence by geography

Another aim of this review was to look for any differences relative to geography. Looking at the seven cardiovascular conditions studied in 3 or more countries, the highest prevalence of MI was recorded in the USA as 19.5%. This figure appears unusually high and may be the result of a relatively small sample size of 113. This result aside, there appears to be a marked increase in prevalence of MI in the UK, recorded at 5.4% and 2.8%, with the other studies investigating Taiwan, the Netherlands and the USA all having similar smaller rates in the range 1.3% to 2.0%. This trend was repeated for CVA, with prevalence of 4.9% and 9.5% reported in the UK compared with 1.6% in the Netherlands.

It was difficult to identify any reliable patterns when analysing the results by country. This may be down to difference in study design and methodology which in turn impacts what measurements are recorded and how that data is reported. The result is that not all the data for each cardiovascular disease is comparable to each other. This coupled with the general lack of studies present within the literature means interpreting trends between countries is challenging.

### Clinical implications

Previous efforts to estimate cardiovascular risk in gout patients have found that when stratified using a risk assessment tool, 56.3% had their cardiovascular risk upgraded after undergoing a carotid ultrasound to assess for the presence of atheromatous plaques [50]. Another study found that after adding gout as a risk factor for cardiovascular events to the risk assessment tool, 38.3% of patients had their risk upgraded [51]. New classification tools may be required to better evaluate the cardiovascular implications of gout. Additionally, raised awareness could allow for more screening for the risk factors of increased prevalence, such as diabetes and hypertension. This intervention could be promoted alongside several other rheumatic conditions, such as psoriatic arthritis or axial spondyloarthritis, to nurture the understanding that the association between rheumatic conditions and cardiovascular diseases exists beyond that of just the well-established link with rheumatoid arthritis.

Furthermore, these results are in line with other studies which have shown an increased risk for sufferers of hyperuricaemia for both MI and CVA [6, 7, 52]. With the current understanding of the underlying process limited, whether synergistic or exclusive, it appears gout and hyperuricaemia have a detrimental effect on the cardiovascular system. Several studies have made an association between serum uric acid and metabolic syndrome [53, 54], suggesting it may have homogenous actions, such as activation of the sympathetic nervous system, renin-angiotensin system and increased levels of pro-inflammatory adipokines and cytokines, which confer an elevated cardiovascular risk through factors including raised heart rate, circulating blood volume and vascular resistance [55].

### Limitations

A strength of this review is the broad inclusion of cardiovascular diseases. There do not appear to be any other reviews that have cast their net as wide when examining the cardiovascular impact of gout. While this has resulted in some sparse reporting of some conditions, for example in aortic stenosis, it lays groundwork for future studies to investigate these trends further.

Limitations within the review include the lack of studies, particularly with large sample sizes, which reported prevalence of given cardiovascular diseases in gout populations. This led to some imprecision in the results which manifested as large confidence intervals in the meta-analysis. It is also plausible that patients with gout would visit healthcare professionals more often than a non-gout control. This would present more opportunity to screen for and diagnose cardiovascular disease. This may result in an overestimation of prevalence and explain why nearly all studies found an increased prevalence when compared to non-gout controls [36]. The high heterogeneity seen in the meta-analysis could be due to different types of studies being carried out in different settings, leading to more uncertainty in the pooled prevalence estimates.

### Conclusion

In summary, this systematic review and meta-analysis highlights the increased prevalence of numerous cardiovascular diseases amongst patients with gout. These results do well to establish a pooled prevalence for several conditions, particularly MI and VTE. This draws attention to the challenge for clinicians to be more vigilant of an increased cardiovascular burden in gout patients. Future research is needed to investigate the link between gout, hyperuricaemia and increased cardiovascular risk and also to establish a more thorough picture of prevalence for the wide variety of cardiovascular diseases.

## Supplementary Information

Below is the link to the electronic supplementary material.

Supplementary file1 (DOCX 351 KB)
