# Supplementary material for: The incidence and prevalence of cardiovascular diseases in gout: a systematic review and meta-analysis
Source: Rheumatol Int. 2021 May 13;41(7):1209–19. doi: 10.1007/s00296-021-04876-6 (PMC8164620; doi:10.1007/s00296-021-04876-6)
Supplement: Supplementary file 1 — Supplementary file1 (DOCX 351 KB) [file 296_2021_4876_MOESM1_ESM.docx]

Supplementary materials for

“**The Incidence and Prevalence of Cardiovascular Diseases in Gout: A Systematic Review and Meta-analysis**”

Peter Cox^1^, Sonal Gupta^2^, Sizheng Steven Zhao^3^, David M Hughes^4^

1 Institute of Systems, Molecular and Integrative Biology,

University of Liverpool

Liverpool

UK

2 School of Medicine

University of Liverpool

Liverpool

UK

3 Musculoskeletal Biology, Institute of Life Course and Medical Sciences

University of Liverpool

Liverpool

UK

4 Department of Health Data Science

University of Liverpool

Liverpool

UK

Correspondence to:

Peter Cox

Institute of Systems, Molecular and Integrative Biology
University of Liverpool,
Biosciences Building,
Crown Street,
Liverpool,
L69 7BE

UK

[hlpcox@liverpool.ac.uk](mailto:hlpcox@liverpool.ac.uk)

| **Supplementary Table S1** Quality assessment using a modified Newcastle-Ottawa Scale | | | | | |
| --- | --- | --- | --- | --- | --- |
| Study | Representativeness | Sample size | Gout definition | Ascertainment of cardiovascular disease | Total |
| Abbott, R. D. 1988 | 2 | 0 | 0 | 1 | 3 |
| Chang, K. 2017 | 1 | 0 | 1 | 1 | 3 |
| Chen, S.Y. 2007 | 1 | 0 | 2 | 1 | 4 |
| Chiu, C. C. 2016 | 1 | 0 | 1 | 1 | 3 |
| Clarson, L.E. 2015 | 2 | 1 | 1 | 1 | 5 |
| Colantonio, L. D. 2020 | 1 | 0 | 1 | 0 | 2 |
| Disveld, I. J. M. 2019 | 1 | 0 | 2 | 1 | 4 |
| Essex, M. N. 2017 | 1 | 0 | 1 | 1 | 3 |
| Francis-Sedlak, M. 2020 | 1 | 0 | 1 | 1 | 3 |
| Huang, C. C. 2015 | 2 | 0 | 1 | 1 | 4 |
| Janssens, H.J.E.M. 2003 | 2 | 1 | 1 | 1 | 5 |
| Janssens, H.J.E.M. 2017 | 2 | 0 | 1 | 1 | 4 |
| Kim, S.C. 2016 | 2 | 0 | 1 | 1 | 4 |
| Kubota, Y. 2016 | 2 | 0 | 0 | 1 | 3 |
| Kuo, C.F. 2010 | 1 | 0 | 0 | 1 | 2 |
| Kuo, C.F. 2013 | 1 | 0 | 1 | 1 | 3 |
| Li, L. 2020 | 2 | 0 | 2 | 1 | 5 |
| Lin, J.C. 2015 | 1 | 0 | 2 | 1 | 4 |
| Meek, I.L. 2013 | 1 | 0 | 2 | 0 | 3 |
| Pan, A. 2015 | 2 | 0 | 0 | 0 | 2 |
| Raisch, D.W. 2009 ABSTRACT | 2 | 0 | 1 | 1 | 4 |
| Seminog, O. O. 2013 | 1 | 0 | 1 | 1 | 3 |
| Singh, J.A. 2018 AF | 1 | 0 | 1 | 1 | 3 |
| Singh, J.A. 2018 MI | 1 | 0 | 1 | 1 | 3 |
| Stack, A.G. 2013 | 2 | 0 | 0 | 1 | 3 |
| Sultan, A. A. 2019 | 2 | 0 | 1 | 1 | 4 |
| Representativeness: 2 for population or primary care level data, 1 hospital, 0 more selective. Sample size: 1 if justified, 0 if not. Gout definition: 2 for classification criteria or physician diagnosis; 1 for diagnostic codes; 0 if self-reported. Ascertainment of cardiovascular disease: 1 if diagnostic code; 0 if self-reported. 0 is given if unclear or unreported. | | | | | |

**Supplementary Fig S1** Distribution of Newcastle-Ottawa Scale bias scores from Table S1

| **Supplementary Table S2** Complete data extraction of studies included in the systematic review | | | | | | | | | |
| --- | --- | --- | --- | --- | --- | --- | --- | --- | --- |
| Study | Country | Data source | Sample Size | Gout diagnosis | Mean Age | Male% | Cardiovascular diagnosis | Prevalence | Incidence per 1000 years |
| **Myocardial infarction** | | | | | | | | | |
| Abbott, R. D. 1988 | USA | Framingham Study | 113 | Self-reported | N/A | 83.2 | Physician diagnosed | 19.47 |  |
| Chen, S.Y. 2007 | Taiwan | Ho-Ping Gout Database, inpatients | 22,572 | Wallace criteria | N/A | 91.2 | Cardiologist reviewed resting ECG for pathological Q-wave in 2 adjacent leads | 1.74 |  |
| Essex, M. N. 2017 | USA | Humedica database | 65,329 | ICD code | 63.7 | 73.2 | ICD code | 1.71 |  |
| Janssens, H.J.E.M. 2017 | Netherlands | GP Database | 1859 | International Classification of Primary Care (ICPC) | 62.5 | 66.9 | ICPC | 2.04 |  |
| Kuo, C.F. 2013 | Taiwan | Taiwanese National Health Insurance database | 26,556 | ICD code | 55.4 | 70.3 | ICD code | 1.74 | 2.2 |
| Seminog, O. O. 2013 (HES) | UK | Hospital Episode Statistics for England | 202,033 | ICD code | 70.3 | 74 | ICD code | 5.44 |  |
| Seminog, O. O. 2013 (ORLS) | UK | Oxford Record Linkage Study | 3,174 | ICD code | 68.8 | 73 | ICD code | 2.8 |  |
| Singh, J.A. 2018 MI | USA | Medicare 5% claims data | 94,809 | ICD code | N/A | N/A | ICD code | 1.32 | 4.1 |
| Clarson, L.E. 2015 | UK | Clinical Practice Research Datalink | 8,386 | Diagnostic code | 66.3 | 69.4 | Medical code |  | M 9.27, F 6.11 |
| **Cerebrovascular accident** | | | | | | | | | |
| Janssens, H.J.E.M. 2017 | Netherlands | GP Database | 1859 | ICPC | 62.5 | 66.9 | ICPC | 1.56 |  |
| Seminog, O. O. 2013 (HES) | UK | Hospital Episode Statistics for England | 202,033 | ICD code | 70.3 | 74 | ICD code | 4.93 |  |
| Seminog, O. O. 2013 (ORLS) | UK | Oxford Record Linkage Study | 3,174 | ICD code | 68.8 | 73 | ICD code | 9.45 |  |
| Clarson, L.E. 2015 | UK | Clinical Practice Research Datalink | 8,386 | Diagnostic code | 66.3 | 69.4 | Medical code |  | M 7.45, F 13.71 |
| Colantonio, L. D. 2020 | USA | REGARDS cohort | 187 | ICD code | 73.8 | 65.8 | Physician diagnosed |  | 9.3 |
| **Heart failure** | | | | | | | | | |
| Essex, M. N. 2017 | USA | Humedica database | 65,329 | ICD code | 63.7 | 73.2 | ICD code | 5.41 |  |
| Francis-Sedlak, M. 2020 | USA | Humanan Research Database | 6831 | ICD code | 71.8 | 67.4 | Diagnostic code | 22.82 |  |
| Janssens, H.J.E.M. 2017 | Netherlands | GP Database | 1859 | ICPC | 62.5 | 66.9 | ICPC | 1.67 |  |
| Lin, J.C. 2015 | Taiwan | N/A | 108 | Wallace and ACR criteria | 58.2 | 90 | Transthoracic echocardiogram | 23.15 |  |
| Colantonio, L. D. 2020 | USA | REGARDS cohort | 187 | ICD code | 73.8 | 65.8 | Physician diagnosed |  | 13.1 |
| **Venous thromboembolism** | | | | | | | | | |
| Chiu, C. C. 2016 | Taiwan | Taiwanese National Health Insurance database | 35,959 | ICD code | 54.7 | 73.7 | ICD code | 1.02 | 1.348 |
| Kubota, Y. 2016 | USA | Atherosclerosis Risk in Communities Study | 647 | Self-reported | N/A | N/A | ICD code | 6.18 |  |
| Raisch, D.W. 2009 ABSTRACT | USA | Veterans Affairs national database | 156,809 | ICD code | N/A | N/A | ICD code | 1.62 |  |
| Li, L. 2020 | Canada | Population Data BC Database | 130,708 | Physician diagnosed | 58.98 | 64.38 | ICD code and prescription of anticoagulation therapy | 1.58 | 2.63 |
| Sultan, A. A. 2019 | UK | Primary Care and NHS Hospital databases | 62,234 | Medical code | 62.4 ± 15.1 | 73.8 | Medical code | 2.38 | 3.73 |
| Huang, C. C. 2015 | Taiwan | National Health Insurance Research database | 57,981 | ICD code | 52.5 | 73.9 | ICD code |  | 0.526 |
| **Cardiovascular mortality** | | | | | | | | | |
| Disveld, I. J. M. 2019 | Netherlands | Gout Arnhem-Liemers Cohort | 700 | Crystal-proven joint fluid analysis | 62 | 81.9 | ICD code |  | 7.71 |
| Kuo, C.F. 2010 | Taiwan | Health screening programme in Chang Gung Memorial Hospital | 1,311 | Medical code or self-report | N/A | N/A | ICD code |  | 2.09 |
| Stack, A.G. 2013 | USA | Third National Health and Nutrition Examination Survey | 468 | Self-reported | N/A | N/A | ICD code |  | 23.1 |
| **Hypertension** | | | | | | | | | |
| Essex, M. N. 2017 | USA | Humedica database | 65,329 | ICD code | 63.7 | 73.2 | ICD code | 17.79 |  |
| Francis-Sedlak, M. 2020 | USA | Humanan Research Database | 6831 | ICD code | 71.8 | 67.4 | Diagnostic code | 84.22 |  |
| Meek, I.L. 2013 | Netherlands | Arthritis Center Twente (outpatients) and the Doetinchem Cohort | 129 | Attending rheumatic outpatients | N/A | 89.9 | Physical examination of BP>140/90 mmHg | 83 |  |
| Pan, A. 2015 | China | Singapore Chinese Health Study | 573 | Self-reported | 58.5 | 60.4 | Self-reported |  | 52.54 |
| **Angina** | | | | | | | | | |
| Abbott, R. D. 1988 | USA | Framingham Study | 113 | Self-reported | N/A | 83.2 | Physician diagnosed | 20.35 |  |
| Janssens, H.J.E.M. 2017 | Netherlands | GP Database | 1859 | ICPC | 62.5 | 66.9 | ICPC | 0.97 |  |
| Clarson, L.E. 2015 | UK | Clinical Practice Research Datalink | 8,386 | Diagnostic code | 66.3 | 69.4 | Medical code |  | M 11.80, F 12.32 |
| **Transient Ischaemic Attack** | | | | | | | | | |
| Janssens, H.J.E.M. 2017 | Netherlands | GP Database | 1859 | ICPC | 62.5 | 66.9 | ICPC | 0.81 |  |
| Clarson, L.E. 2015 | UK | Clinical Practice Research Datalink | 8,386 | Diagnostic code | 66.3 | 69.4 | Medical code |  | M 6.04, F 9.34 |
| **Atrial Fibrillation** | | | | | | | | | |
| Francis-Sedlak, M. 2020 | USA | Humanan Research Database | 6831 | ICD code | 71.8 | 67.4 | Diagnostic code | 21.48 |  |
| Kim, S.C. 2016 | USA | United HealthCare database | 70,015 | ICD code | 56.8 | 81.4 | ICD code | 1.47 | 7.19 |
| Singh, J.A. 2018 AF | USA | Medicare 5% claims data | N/A | ICD code | N/A | N/A | ICD code |  | 43.4 |
| **Peripheral Vascular disease** | | | | | | | | | |
| Janssens, H.J.E.M. 2017 | Netherlands | GP Database | 1859 | ICPC | 62.5 | 66.9 | ICPC | 1.51 |  |
| Clarson, L.E. 2015 | UK | Clinical Practice Research Datalink | 8,386 | Diagnostic code | 66.3 | 69.4 | Medical code |  | M 5.60, F 7.09 |
| **Any Coronary Heart Disease** | | | | | | | | | |
| Clarson, L.E. 2015 | UK | Clinical Practice Research Datalink | 8,386 | Diagnostic code | 66.3 | 69.4 | Medical code |  | M 28.46, F 9.11 |
| Colantonio, L. D. 2020 | USA | REGARDS cohort | 187 | ICD code | 73.8 | 65.8 | Physician diagnosed |  | 16.0 |
| **Aortic Stenosis** | | | | | | | | | |
| Chang, K. 2017 | USA | Outpatients | 112 | Medical Records | 80.1 | 99.1 | Transthoracic echocardiogram | 21.43 |  |
| **Aortic aneurysm** | | | | | | | | | |
| Janssens, H.J.E.M. 2017 | Netherlands | GP Database | 1859 | ICPC | 62.5 | 66.9 | ICPC | 0.91 |  |
| **Cardiovascular disease** | | | | | | | | | |
| Janssens, H.J.E.M. 2003 | Netherlands | Continuous Morbidity Registration | 170 | Medical code | N/A | 91 | Diagnostic code | 25.88 |  |

F, Female; HES, Hospital Episode Statistics; ICD, International Classification of Diseases; ICPC, International Classification of Primary Care; M, Male; ORLS, Oxford Record Linkage Study.

Funnel plots for each meta-analysis


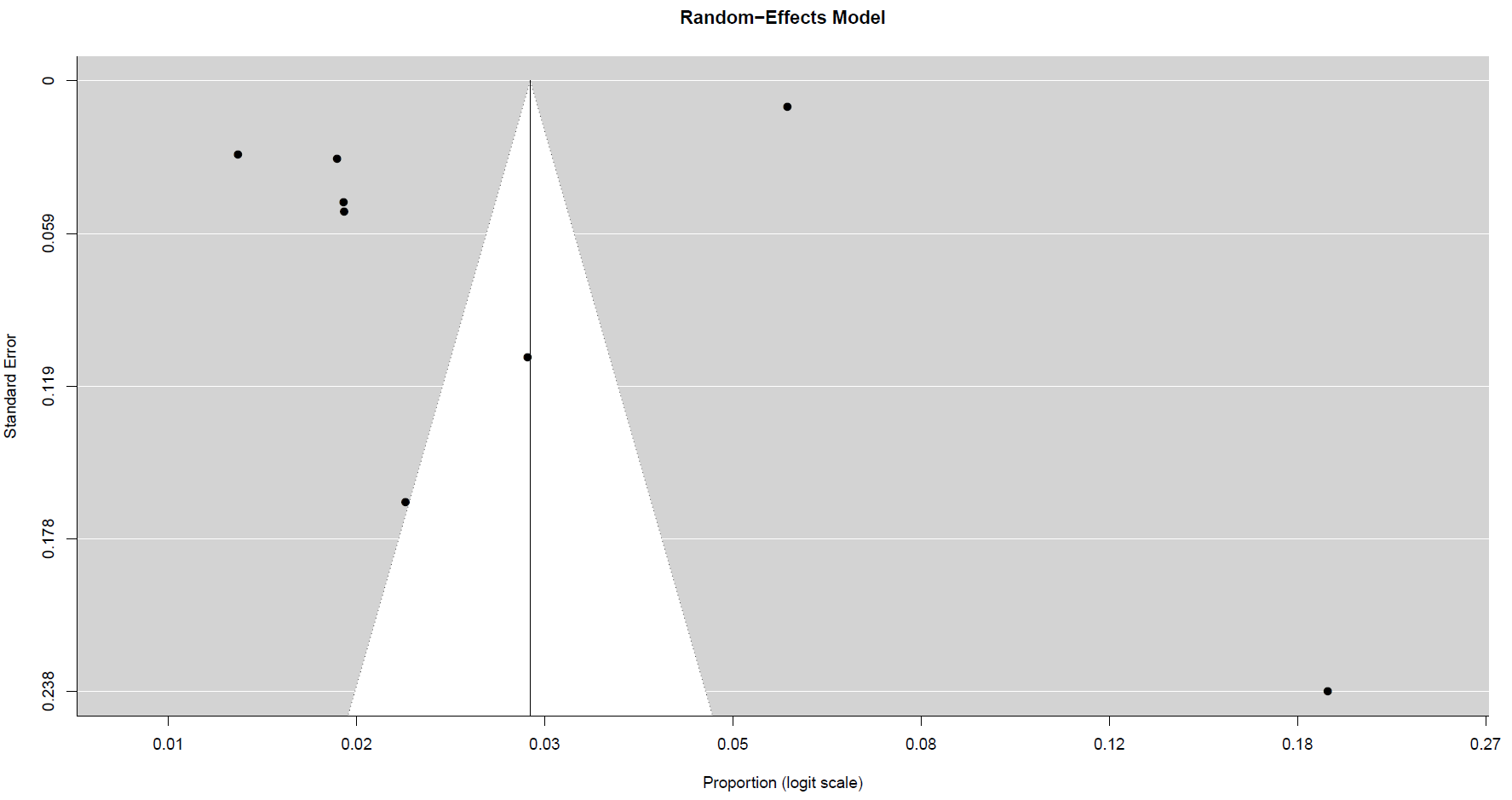


**Supplementary Fig S2** Funnel plot for pooled prevalence of myocardial infarction


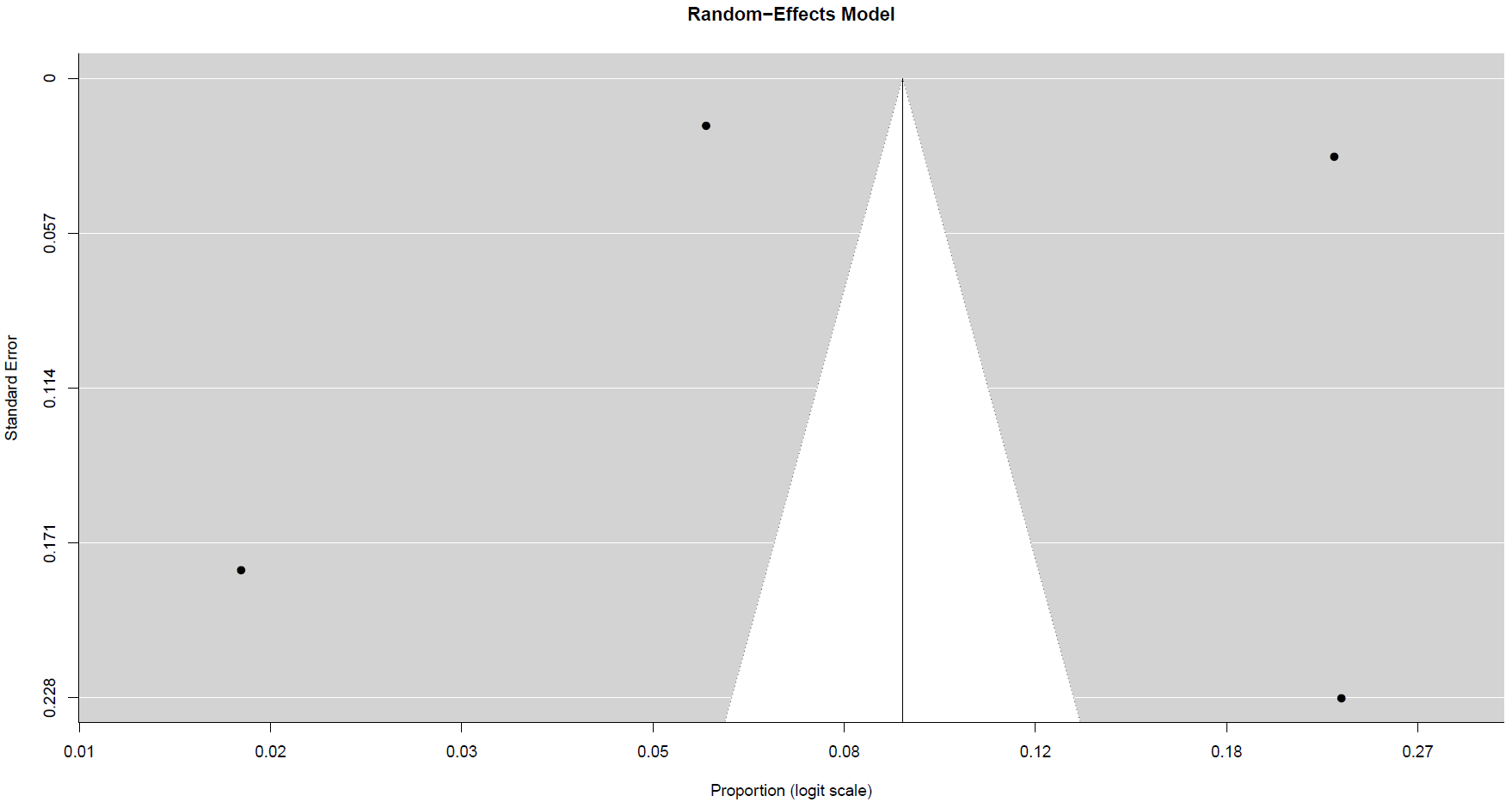


**Supplementary Fig S3** Funnel plot for pooled prevalence of heart failure


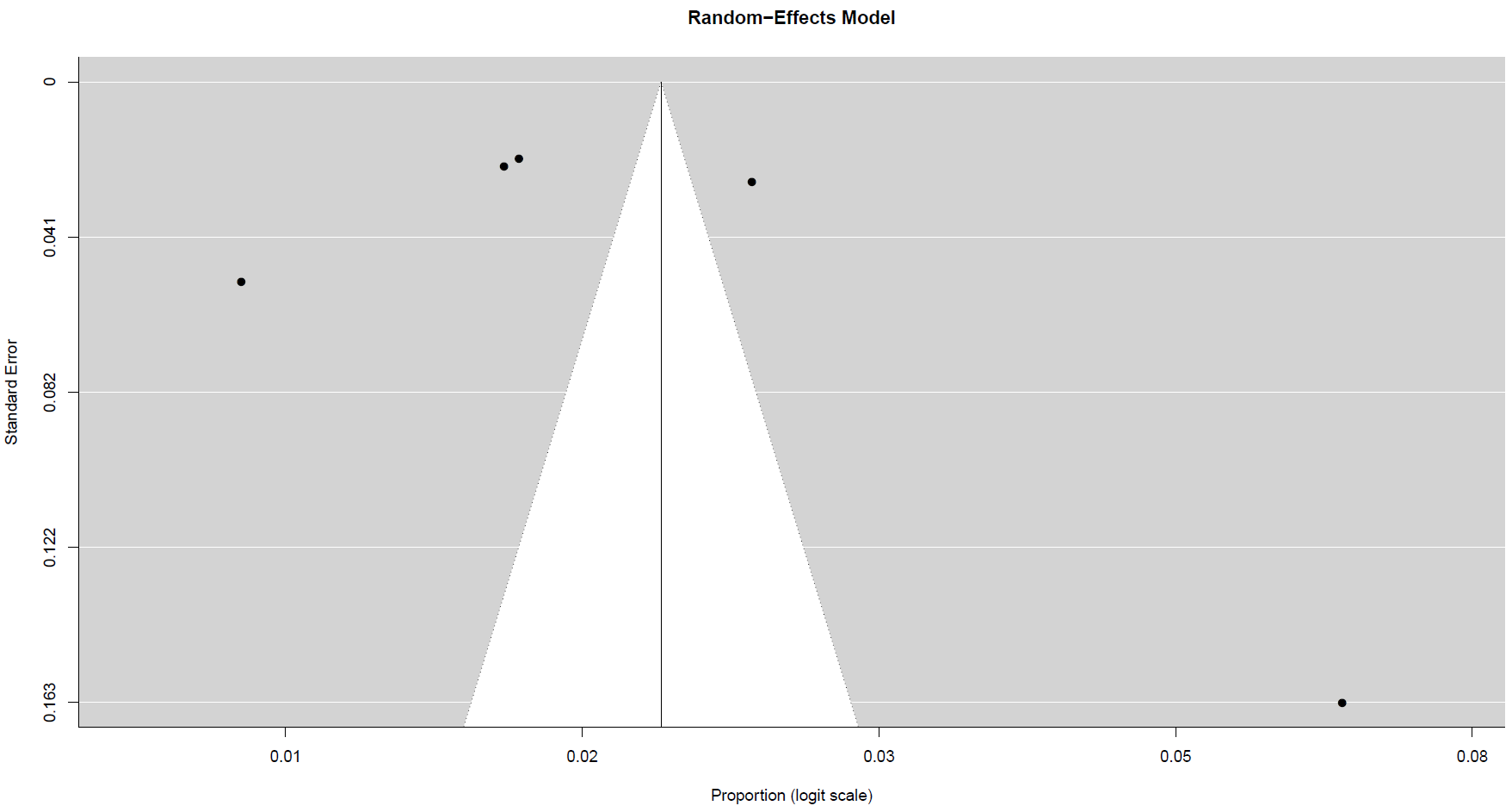


**Supplementary Fig S4** Funnel plot for pooled prevalence of venous thromboembolism


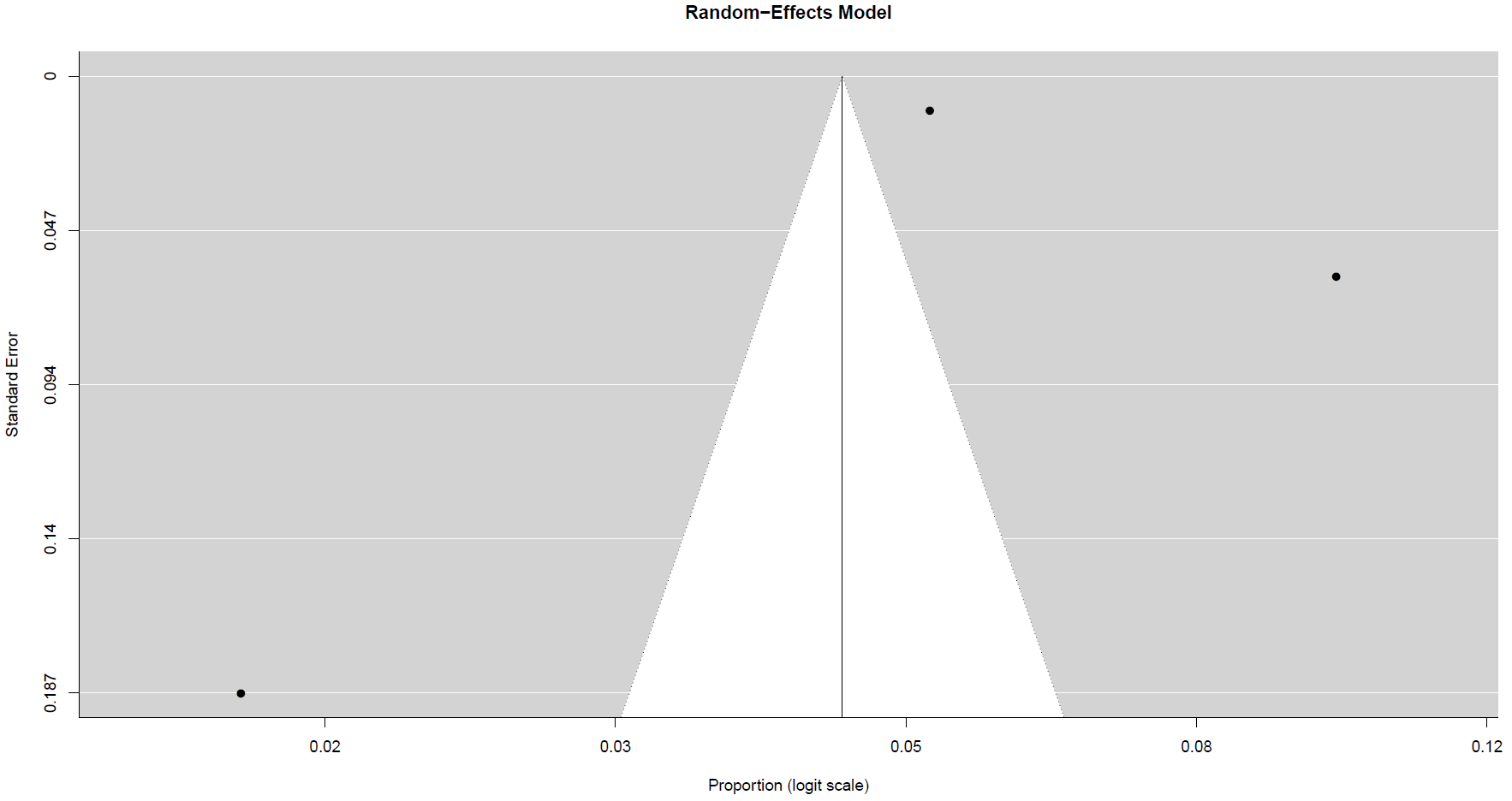


**Supplementary Fig S5** Funnel plot for pooled prevalence for cerebrovascular accident


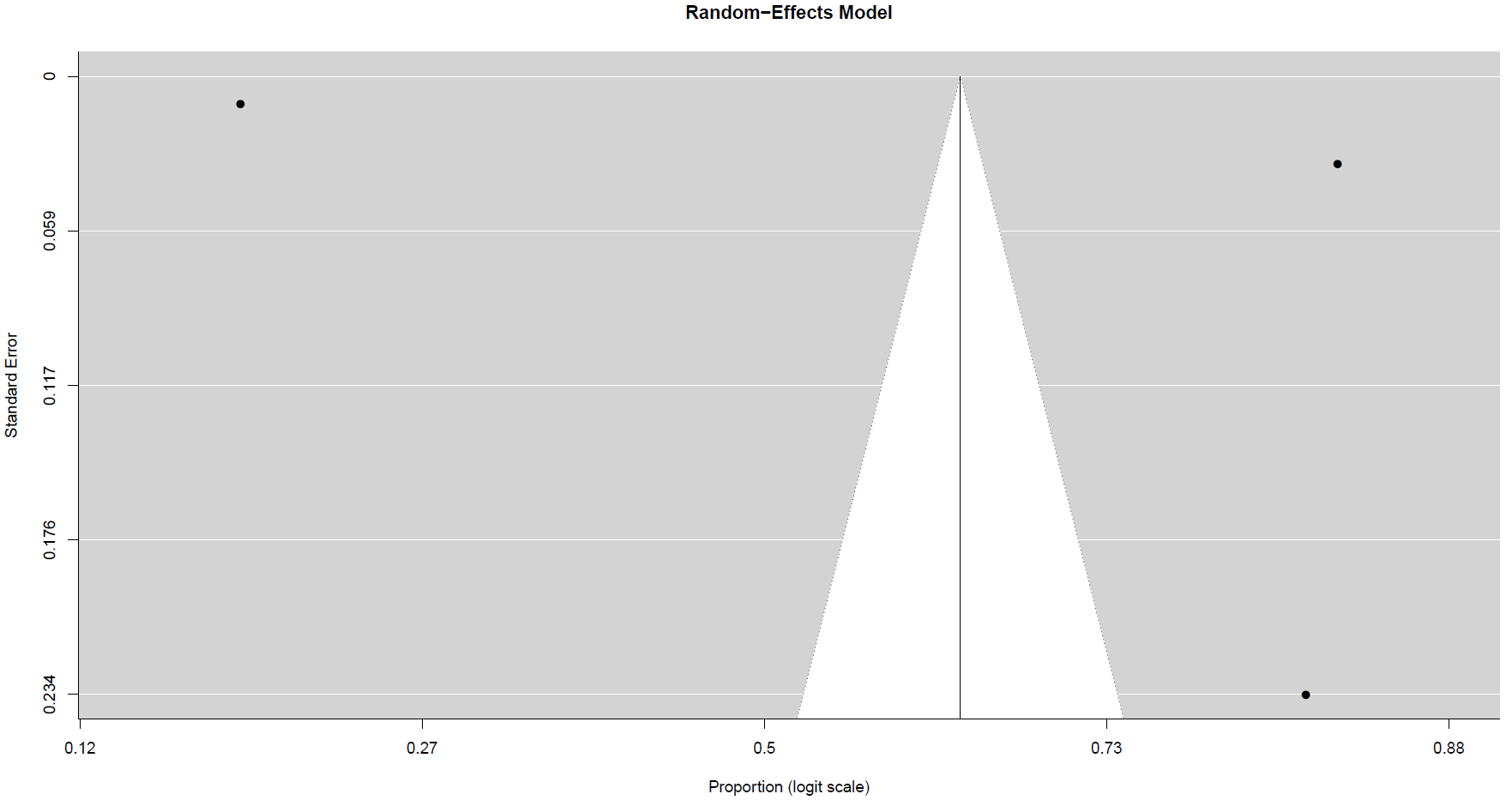


**Supplementary Fig S6** Funnel plot for pooled prevalence for hypertension
